# Disentangling bacterial diversity and biogeography in snow-covered regions

**DOI:** 10.1007/s11274-026-04918-w

**Published:** 2026-04-28

**Authors:** Jéssica Bianca da Silva, Paulo Eduardo Aguiar Saraiva Câmara, Luiz Henrique Rosa, Valéria Maia Oliveira

**Affiliations:** 1 Division of Microbial Resources, Research Center for Chemistry, Biology and Agriculture (CPQBA), UNICAMP, Alexandre Cazellato Ave., Betel, São Paulo, Paulínia 13148-218 Brazil; 2 Institute of Biology, State University of Campinas, Campinas, SP CEP: 13083-862 Brazil; 3 Department of Botany, University of Brasilia – UNB, Brasilia, Brazil; 4 Institute of Biological Sciences, Federal University of Minas Gerais - UFMG, Belo Horizonte, MG CEP 31270-901 Brazil

**Keywords:** Microbial biogeography, Snow microbiome, Antarctica, Cold environments, Supraglacial ecosystems

## Abstract

This study investigated the bacterial diversity of snow-inhabiting microbial communities across multiple geographic locations, including Antarctic and temperate regions. Using high-throughput sequencing of the 16S rRNA gene from Antarctic snow samples and comparisons with publicly available datasets from other cold regions worldwide, we assessed patterns of taxonomic diversity and the influence of geographic and environmental factors on snow bacterial communities. Our results revealed that bacterial communities from Martel Inlet (King George Island) exhibited lower diversity compared to other Antarctic sites, likely influenced by the geographic characteristics. In contrast, snow microbial communities from temperate regions such as Austria, Quebec, and Iceland showed higher diversity, potentially driven by overlapping environmental conditions including temperature range, snow dynamics, and seasonal variability. Network analyses revealed distinct interaction patterns among regions, with more dynamic and competitive microbial networks observed in Maritime Antarctic environments, while continental snow ecosystems exhibited more compartmentalized and stable network structures. Overall, our findings highlight the combined influence of geographic distance and environmental conditions in shaping microbial diversity and ecological interactions in snow ecosystems across different regions of the globe.

**Supplementary Information:**

The online version contains supplementary material available at 10.1007/s11274-026-04918-w.

## Introduction

The cryosphere is a vital component of the Earth’s surface, acting as a key regulator of the global climate by storing freshwater, influencing ocean circulation, and reflecting solar radiation. It encompasses regions characterized by the presence of frozen water and soil. These include permafrost, where the ground remains permanently frozen, as well as areas of frozen water such as sea ice, glaciers, icebergs, polar ice caps, and seasonal snowpack (Krissek and St. John 2025; Spiridonov et al. 2025; Williams et al. 2025). Seasonal snow, in particular, blankets more than one-third of the Earth’s surface, making it the most prevalent element within the cryosphere (Fair et al. 2025; Slatyer et al. 2022).

Snow-covered landscapes are widely distributed across various regions of our planet. These include the Arctic region, encompassing extensive territorial areas of Europe, northern Asia, and North America, high-altitude mountain ranges and temperate regions during the winter season, such as the Arctic tundra, boreal forests, and alpine ecosystems (Stuefer et al. 2025; Thomas 2022), as well as the vast polar expanses of Antarctica and sub-Antarctic islands in the Southern Hemisphere. King George Island, the largest island of the South Shetland Archipelago in maritime Antarctica, is largely dominated by an ice cover, corresponding to approximately 92% of its extension (Francis et al. 2026; Lim et al. 2022a). The climate dynamics in this region is characterized by the influence of cyclonic systems, which transport masses of warm and humid air, resulting in energetic winds and a notable amount of precipitation. Previous records in the area of the island have revealed an average annual rainfall of 598.2 mm and a broad variation (2–73 cm) in the depth of the snow cover (Lim et al. 2022a; Lorenz et al. 2023).

Seasonal snow plays a crucial role in regulating global climate patterns and has a significant influence on local ecosystems. Snow extent and persistence drive the occurrence of freeze–thaw events, and can have a profound impact on the composition of the soil microbial community and biogeochemical cycles mediated by microorganisms (Hua et al. 2024; Ji et al. 2022). Snow cover can be regarded as a dynamic habitat that mediates the interactions among microorganisms, nutrients, atmosphere, and soil (Xu et al. 2024; Yin et al. 2024).

Despite the apparent hostility of snow ecosystems, as low temperatures, atmospheric low humidity and limited liquid water availability, in addition to high UV radiation levels during summer, they harbor diverse and active microbial communities that can be endemic or transported from distant locations. Snow serves as a dynamic reservoir of nutrients and microorganisms due to atmospheric deposition of non-indigenous microorganisms and particulate organic matter (Duan et al. 2025; Kosolapova et al. 2025). These microorganisms are adapted to extreme conditions and play a key role in surface soil biogeochemical cycles and in interactions supporting inter-trophic dependence and ecosystem functioning. Understanding the composition, structure and distribution of snow microbiomes and their environmental drivers can shed light into their evolution, ecological roles and the potential impacts of climate change over the ecosystem functioning in snow covered regions. Microbial biogeography is essential to unveiling how these microorganisms are distributed across different snow regions, identifying endemic species, and predicting their responses to environmental and climatic changes. Studies of snow as an ecosystem have increasingly addressed the structure and diversity of microbial communities (Baloh et al. 2019; Sanchez-Cid et al. 2022, 2022; Villeneuve et al. 2023; Winkel et al. 2022; Yakimovich et al. 2020). However, research specifically focused on microbial biogeography in snow remains incipient. Recent studies have investigated snow microbial communities at local and regional scales within regions or countries. These include studies from the East Antarctic Plateau and Maritime Antarctica (Parro et al. 2025; Soto et al. 2022), Arctic and sub-Arctic environments such as Svalbard, Greenland and Iceland (Keuschnig et al. 2023; Lutz et al. 2016), and temperate mountain regions such as Colorado (Honeyman et al. 2018). Microbial community studies in high-altitude mid-latitude mountain systems have also been conducted in the Alps, Himalayas, Karakoram, and Eastern Anatolia (Azzoni et al. 2018). In addition, other studies reported intercontinental contrasts, including comparisons between Fennoscandia and Colorado and between the Swiss and Australian Alps (Brown and Jumpponen 2019; Wunderlin et al. 2016) (Brown and Jumpponen 2019; Wunderlin et al. 2016). Despite these efforts, no study has hitherto conducted a biogeographical analysis directly comparing Antarctic snow microbiomes with those from sub-Arctic and temperate nival environments.

Antarctica is the coldest, windiest, and driest continent on Earth, imposing some of the most extreme conditions for life. The Antarctic continent is commonly divided into maritime and continental ecological zones. Maritime Antarctica comprises the western coastal regions and nearby offshore islands of the Antarctic Peninsula, including the South Shetland archipelago. The marine influence provides positive temperatures in summer, with a range from − 10 to + 2 °C in the warmer months (November to March) (Bölter et al. 2002; Convey 2013; Silva et al. 2023). King George Island, located in the South Shetland archipelago, represents a low-altitude coastal environment characterized by high atmospheric humidity and frequent precipitation. Meteorological records already indicated an average annual air temperature of − 2.5 ± 4.6 °C, a mean relative humidity of 87.1 ± 7.8%, and average annual precipitation of approximately 598 mm (Lim et al. 2022b).

In contrast, Continental Antarctica represents one of the driest regions on Earth, exhibiting some of the lowest precipitation rates globally (Nicola et al. 2023; Varliero et al. 2024). The Antarctic Plateau, on the East Antarctica, has recorded the lowest air temperature ever measured on Earth’ surface at Vostok Station (− 89.2 °C, but it can reach − 93.2 °C) (Parro et al. 2025). In addition to extremely low temperatures, the Antarctic Plateau is characterized by low absolute humidity, high levels of UV radiation during the austral summer, scarcity of liquid water, and oligotrophic conditions. Due to its proximity to the South Pole, the atmospheric water-vapor content over the Antarctic Plateau is exceptionally low, with mean annual values ranging between 200 and 700 precipitable micrometers (µm) (Genthon et al. 2022; Parro et al. 2025).

Here, we present an investigation into the drivers of snow-borne bacterial communities across seasonal snowpacks, including samples collected along a latitudinal gradient in the Maritime Antarctica as well as from the Antarctic Plateau (Antarctic Continent). Additionally, we performed an intercontinental analysis to examine global similarities and differences in snow-borne bacterial communities by comparing Antarctic samples with those from Canada, Poland, Austria, and Iceland. Specifically, we hypothesize that (1) microbial diversity in Antarctica is lower compared to temperate snow-covered regions due to the extreme isolation and environmental conditions; (2) snow-inhabiting bacterial communities will vary significantly along the latitudinal gradient, both regionally (within Antarctic regions) and globally (between Antarctic and non-Antarctic regions).

## Materials and methods

### Research area

King George Island (KGI), the largest island of the South Shetland archipelago, is located at approximately 63° S, north of the Antarctic Peninsula in the Maritime Antarctica region. Around 90% of the island’s surface is covered by ice. Its climate is influenced by cyclonic systems that bring warm, humid air, strong winds, and high precipitation, typical features of a maritime Antarctic environment (Evangelista et al. 2024; Lorenz et al. 2023; Rojas-Macedo et al. 2025).

Admiralty Bay, the largest bay on KGI, has three inlets: Mackellar and Martel in the northern portion, and Ezcurra in the western portion. Martel Inlet is a semi-enclosed fjord bordered by tidewater glaciers and a terrestrial shoreline. The inlet also hosts the Comandante Ferraz Brazilian Research Station and is a research area of special interest for the Brazilian Antarctic Program (PROANTAR) (Gheller and Corbisier 2022; Perondi et al. 2022).

### Snow sampling

**Fig. 1:**
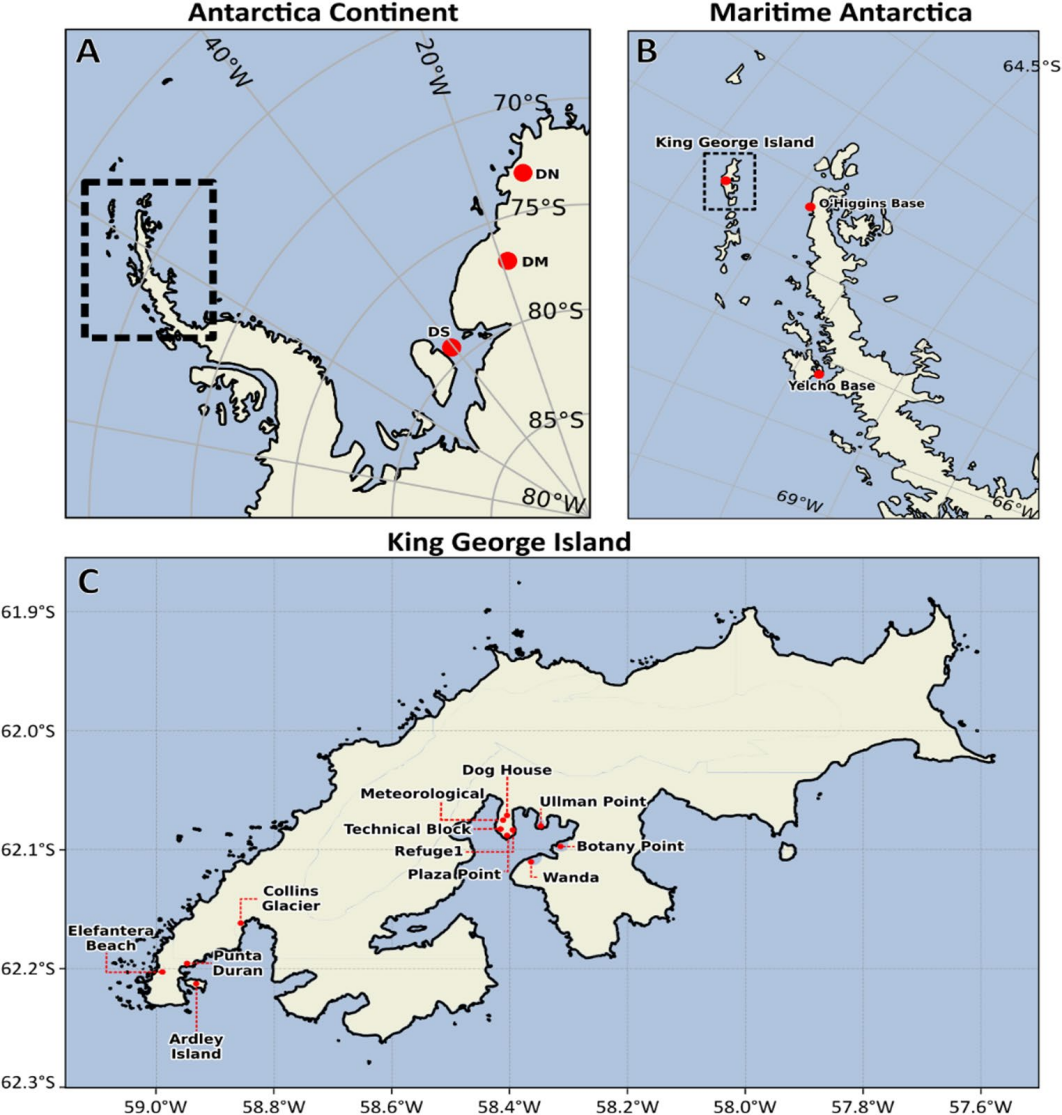
(**A**) Map of the East Antarctic Plateau, showing the location of the three main sampling sites along the snow collection transect. Sampling points are marked with red circles (DN = drill north, DM = drill middle, DS = drill south). The black dashed rectangle indicates the region of the Maritime Antarctica. (**B**) Zoomed-in view of the Maritime Antarctica, highlighting the positions of O’Higgins Base, Yelcho Base, and King George Island (highlighted by a dashed black rectangle), all marked with red points. (**C**) Map of King George Island, showing the sampling sites in Martel Inlet and the Fildes Peninsula (Ardley Island, Collins Glacier, Punta Duran and Elefantera Beach). Maps were generated using the python libraries cartopy and matplotlib based on publicly available geographic shapefiles

Seasonal snow samples were collected in Martel Inlet (King George Island, 62°04′ S, 58°21′ W), during two Antarctic expeditions organized by the Brazilian Antarctic Program (PROANTAR). During the first expedition (December 2019 – January 2020), samples were taken from five sites: Dog House, Meteorological Station, Ullman Point, Botany Point, and Wanda Glacier (Fig. 1, Table S1). In the second expedition, held in November 2021, samples were collected from Plaza Point, Refuge 1, and Technical Block sites (Fig. 1, Table S1). At each site, snow was collected to a depth of approximately 50 cm using a sterile shovel cleaned with 70% ethanol before each sampling. Snow from each site was placed into sterile 20 L buckets, transported to the microbiology laboratory at Comandante Ferraz Station, and allowed to thaw completely. Thawed samples were filtered through 0.22 μm membranes using a three-cup manifold (250 mL) until saturation. The membranes were then transferred to Petri dishes, sealed with parafilm, and stored at − 80 °C until DNA extraction. The same sampling and filtration procedures were applied consistently across both expeditions.

### DNA extraction from antarctic snow samples

Total DNA was extracted directly from the filter membranes used to concentrate microbial cells from the snow samples. DNA extractions were performed using the DNeasy PowerSoil Kit (QIAGEN), with specific adaptations to the manufacturer’s protocol as follows: small pieces of the membranes used to filter the snow were soaked in the C1 solution for 3 h at room temperature. The tubes were then incubated at 65 °C for 10 min and subjected to continuous vortex agitation at maximum speed for 10 min. Further, the steps were followed according to the manufacturer’s protocol. The DNA obtained was purified using the One Step PCR Inhibitor Removal kit (Zymo). For the first expedition, snow samples were sent for sequencing to Macrogen Inc. (Seoul, South Korea). The 16S rRNA gene V3–V4 region was amplified using primers Bakt_341F (5′-CCTACGGGNGGCWGCAG-3′) and Bakt_805R (5′-GACTACHVGGGTATCTAATCC-3′), and sequencing was performed on an Illumina MiSeq platform using paired-end 2 × 300 bp reads. For the second expedition, DNA samples were sequenced at NGS Soluções Genômicas (Piracicaba, SP, Brazil). The 16S rRNA V3–V4 region was amplified using primers 341 F (5′-CCTACGGGNGGCWGCAG-3′) and 785R (5′-GACTACHVGGGTATCTAATCC-3′), and sequencing was carried out on an Illumina MiSeq platform with paired-end 2 × 250 bp reads. Negative controls were not included during library preparation.

### 16S rRNA gene amplicon datasets

The global snow microbiome dataset analyzed includes both 16S rRNA gene amplicon sequencing data generated from this study and publicly available datasets. These published datasets cover both Antarctic and non-polar regions, including regions from Austria, Canada, Iceland, and Poland (**Table ****S1**). In Antarctica, the publicly available datasets are derived from Maritime Antarctica (Ardley Island, Collins Glacier, Elefantera Beach, Punta Duran, O’Higgins and Yelcho Base) and the Antarctica Continent (Antarctic Plateau) **(**Fig. 1**)**. The datasets analyzed in this study focus on the V3-V4 region of the 16S rRNA gene. Details regarding the publicly available sequences are provided in **Table ****S1**.

### Bioinformatics

Quality of raw sequences was checked with the FastQC program (Andrews 2010). Then, reads were trimmed with the Trimmomatic tool v 0.36 (Bolger et al. 2014). Amplicon sequences of the 16S rRNA gene were processed using QIIME2 2021.4 (Quantitative Insights Into Microbial Ecology) tool (Bolyen et al. 2019), and denoised by the DADA2 workflow to generate amplicon sequence variants (ASVs) (Callahan et al. 2016). Taxonomic classification of ASVs was performed using the SILVA database v138 (Quast et al. 2013).

Sequences classified as eukaryotes, mitochondria, and chloroplasts were removed from the datasets. To account for differences in sequencing depth, all samples were rarefied to an equal number of 10,000 sequences per sample using the *rarefy_samples* function in the microeco package. Rarefaction curves for all sites were generated using the microeco and phyloseq packages to verify sufficient sampling depth (Liu et al. 2021; McMurdie and Holmes 2013).

### Geographic and climate data

Latitude, longitude and altitude data related to the sequence datasets were obtained from the respective scientific articles. The climate parameters for each sampling year were obtained from the monthly average data from ERA5-Land, a global land-surface dataset that provides hourly estimates of a large number of atmospheric, land and oceanic climate variables (http://cds.climate.copernicus.eu10.24381/cds.68d2bb30). Using the latitude and longitude information, we selected: temperature of snow layer, snowfall, snowmelt, solar radiation and wind velocity for all sites. Detailed metadata are found in Table S1.

### Statistical analyses

All statistical analyses were carried out in the R environment (version 4.3.0) with the help of specific packages for analyzing microbiomes, including phyloseq 1.44.0 (McMurdie and Holmes 2013), vegan (Oksanen et al. 2015) and microeco (Liu et al. 2021). The non-parametric Kruskal-Wallis test was used to identify significant differences in microbial diversity among the different sites. The weighted UniFrac dissimilarity metric (Lozupone et al. 2011) was employed to evaluate both phylogenetic distance and species abundance. The dissimilarities among microbiomes according to the sampling sites were subjected to Principal Coordinates Analysis (PCoA) for visualizing and interpreting community differences. ANOSIM (Analysis of Similarities) was employed to evaluate the differences between groups by comparing the rank-based dissimilarities within and between groups and PERMANOVA, a permutation-based extension of ANOVA (Analysis of Variance), was used to assesses whether the mean composition of multiple groups differs significantly.

Lefse (Linear Discriminant Analysis Effect Size) (Segata et al. 2011) was used to identify microbial groups exhibiting differential abundance across regions. Collectively, these analyses provide insights into the predominance of different microbial groups within each region. Mantel correlation analyses were conducted to investigate the influence of geographic variables on microbial communities. These analyses employed similarity matrices derived from microbiomes and from environmental variables, aiming to determine whether microbial communities are significantly affected by environmental factors. To further evaluate spatial patterns, the distance–decay relationship was assessed by performing linear regression analyses between pairwise geographic distances and microbial community dissimilarity values. This approach was used to test whether community dissimilarity increased with increasing geographic distance, consistent with patterns expected under isolation-by-distance.

Co-occurrence network analysis was performed to explore inferred microbial interactions across the different snow-covered regions. Co-occurrence network was constructed for each region based on the ASV frequency table. Pairwise correlations were inferred using the Python module SparCC (Friedman and Alm 2012), which accounts for compositionality in microbiome data. Only strong (|SparCC| > 0.6) and statistically significant (*p* < 0.01) correlations were retained to construct the adjacency matrix. Topological properties of the resulting networks were then calculated using the MetNet package (Peng Chen 2023). Node roles within the network were determined based on within-module connectivity (Zi) and among-module connectivity (Pi), following the modular cartography framework proposed by Guimerà and Nunes Amaral (2005). According to this framework, nodes were classified using the commonly adopted thresholds of Zi = 2.5 and Pi = 0.62, peripherals (Zi < 2.5, Pi < 0.62), module hubs (Zi ≥ 2.5, Pi < 0.62), connectors (Zi < 2.5, Pi ≥ 0.62), and network hubs (Zi ≥ 2.5, Pi ≥ 0.62) (Olesen et al. 2007; Poudel et al. 2016).

## Results

### Microbial richness and diversity in snow regions

A total of 12,611 bacterial ASVs were obtained from the snow dataset after removing eukaryotic, mitochondrial and chloroplast sequences. Richness estimates (Chao1), alpha diversity indices (Shannon and Simpson), and phylogenetic diversity (PD) were significantly different between groups (Kruskal-Wallis *p* = 0.001) and are detailed in Table S2.

**Fig. 2:**
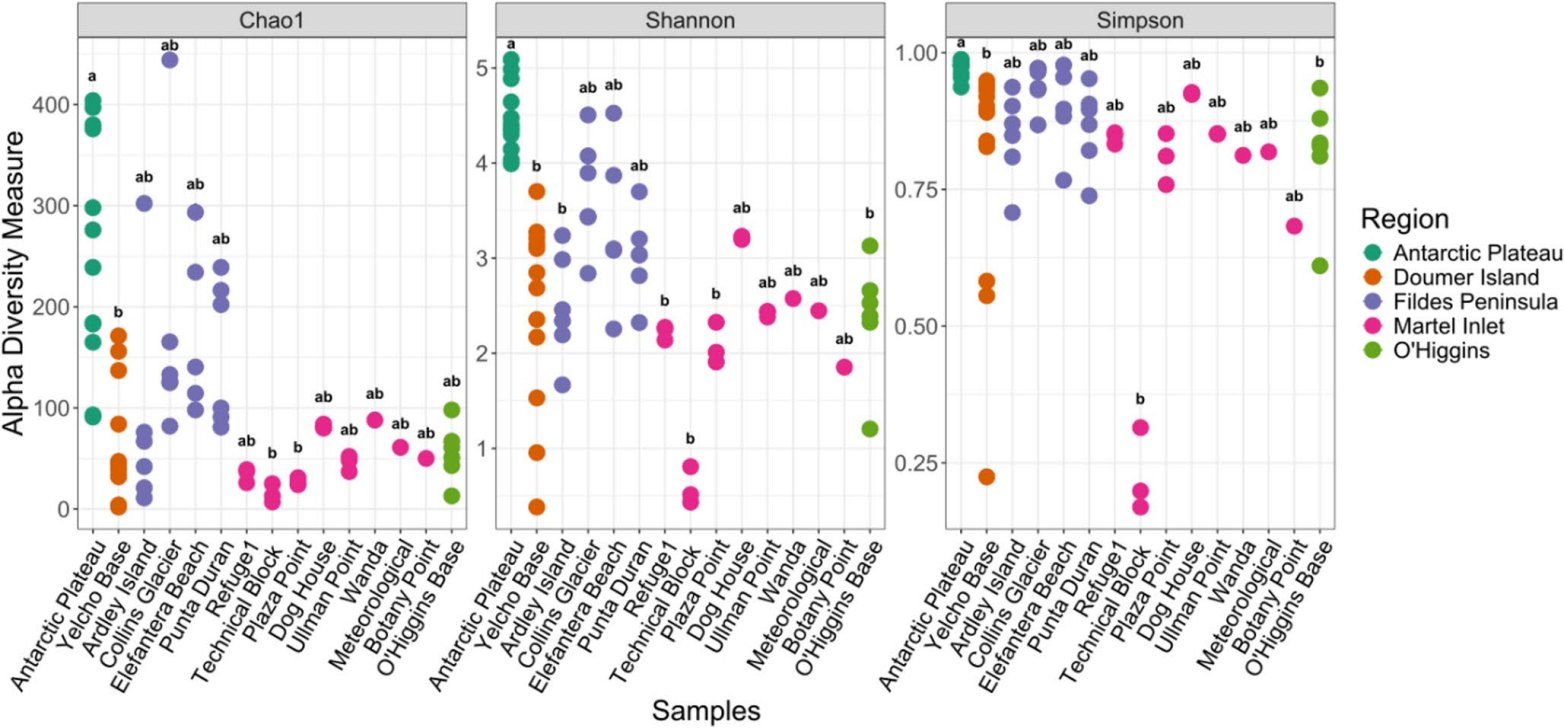
Alpha diversity metrics of bacterial communities in Antarctic sites. The sites have been colored according to the region to which they belong

Among Antarctic sites, post-hoc comparisons (Dunn’s test) revealed that the Continental Antarctica samples (Antarctic Plateau) exhibited the highest richness estimates (Chao1), significantly different from Yelcho Base, Technical Block, and Punta Plaza **(**Fig. 2, Table S2**)**. A similar trend was observed for diversity indices (Shannon, Simpson, and PD), with the Antarctic Plateau presenting significantly higher diversity compared to Ardley Island, Refuge1, Technical Block, Plaza Point, and O’Higgins Base.

Outside Antarctica, Austria sites revealed the highest richness and diversity values, followed by Poland, Iceland and Canada (Table S2). The global analysis revealed the highest richness values in Austria sites, Collins Glacier (Maritime Antarctica) and Antarctic Plateau (number of species > 400), while the lowest values were observed at Technical Block, Ardley Island, O’Higgins Base, Yelcho Base (Maritime Antarctica), and Snaefallsjoekull in Iceland (number of species < 22). Diversity was higher in samples from Austria sites, the Antarctic Plateau and Kociol Malego (Poland). In contrast, some samples from Maritime Antarctica (Technical Block, Yelcho Base), and Iceland exhibited the lowest values for diversity (Table S2).

**Fig. 3:**
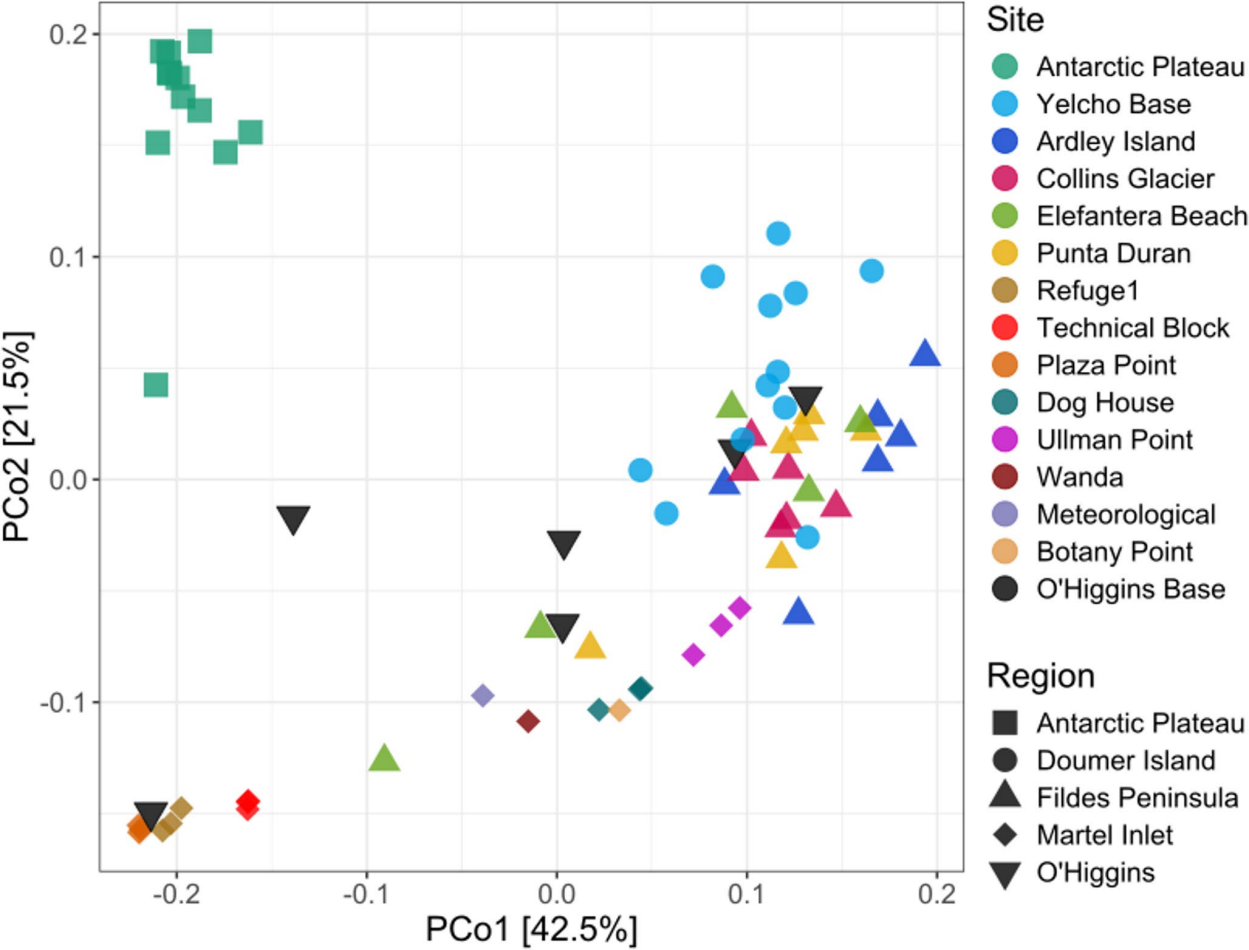
PCoA showing the phylogenetic distance of snow microbiome from different Antarctic sites, based on the Weighted UniFrac metric. PCoA was performed with significative components PC1 and PC2. Sites were color-coded according to the locations of sampling and shaped according to the regions. The Antarctic Plateau was the only Continental Antarctic site analyzed in this study, whereas Doumer Island, Fildes Peninsula, Martel Inlet, and O’Higgins sites represented the Maritime Antarctica region

Beta diversity analysis revealed distinct clustering patterns among bacterial communities from different Antarctic locations (Fig. 3). Samples from the Antarctic Plateau, on the continent, formed a well-defined separate cluster, indicating a highly homogeneous distinct bacterial composition compared to the other sites. Bacterial communities from O’Higgins Base shared similarities with multiple clusters, suggesting inter-site variability. Within Martel Inlet, sites such as Dog House, Ullman Point, Wanda, Meteorological, and Botany Point clustered closely together, reflecting similar bacterial communities. Yelcho Base and Collins Glacier showed interspersed clustering, indicating some degree of shared bacterial composition and abundances. Additionally, Refuge1, Plaza Point, and Technical Block form another distinct cluster, suggesting local bacterial community similarities. These patterns highlight the influence of environmental and geographic factors on bacterial community structure across Antarctic regions.

The significant differences in bacterial composition across the groups were confirmed by ANOSIM (*R* = 0.6837) and PERMANOVA (R² = 0.7366), with a *p*-value of 0.001 indicating statistical significance. This suggests a clear separation between Antarctic sites, with 73.66% of the variation in microbial communities attributable to differences between the sites. The PERMANOVA analysis revealed that season had a significant effect on bacterial community structure (R² = 0.083, F = 6.68, *p* = 0.004), explaining approximately 8% of the total variance, suggesting that seasonal conditions contribute to community differentiation but are not the primary driver compared with spatial and environmental factors.

**Fig. 4:**
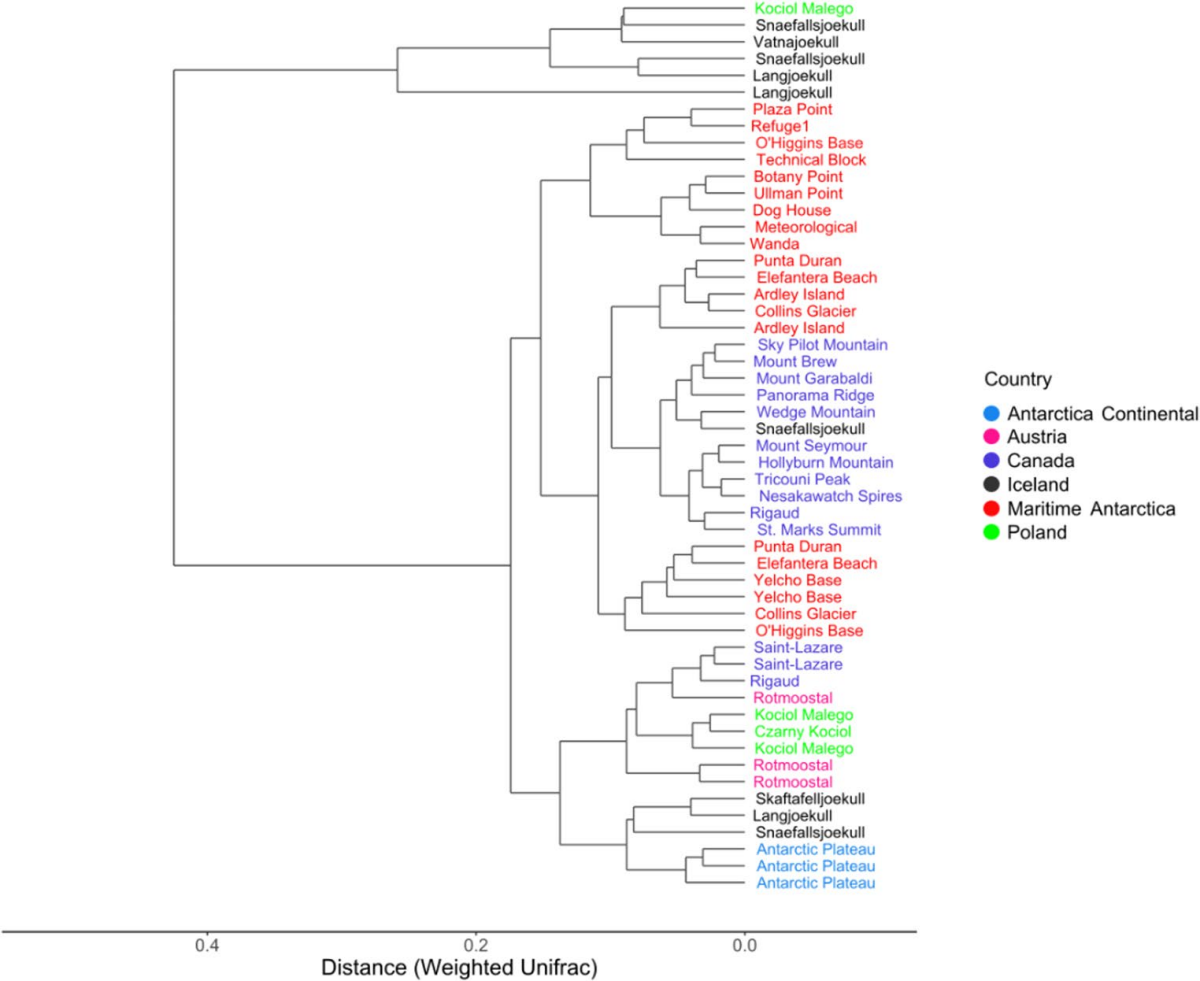
Dendrogram displaying the hierarchical relationship among globally scattered snow bacterial communities based on phylogenetic distance (Weighted Unifrac). Sites were color-coded according to geographic regions

According to the dendrogram analysis (Fig. 4), the samples clustered into four major groups, highlighting a strong phylogenetic divergence among them. One of these branches is primarily represented by Icelandic sites. Although these bacterial communities predominantly originate from samples collected during the summer season (**Table ****S1**), the Icelandic locations exhibited a greater degree of dispersion. A second cluster was formed by samples from Maritime Antarctica and Canadian samples (British Columbia). The bacterial communities from these sites showed a similar phylogenetic pattern with each other. However, a clear divergence between Martel Inlet sites (Plaza Point – Wanda) was noted. Finally, Antarctic Plateau samples clustered separately from the other Antarctic sites, forming a branch that is more closely related to European sites, particularly those from Iceland. Most European samples (from Poland, Austria, and some locations in Iceland) clustered within the same branch as the Antarctic Plateau samples.

The betadisper analysis (F = 5.949, *p* = 0.001) revealed significant group dispersion, suggesting that some sites exhibit greater variability in bacterial composition, possibly due to environmental heterogeneity. The PERMANOVA results confirmed that geographic location is the primary driver of microbial variation (R² = 0.4740, *p* = 0.001), reinforcing the importance of geographic factors. ANOSIM results further supported these findings, showing a high R-value (*R* = 0.7509, *p* = 0.001), indicating strong dissimilarities in bacterial communities between geographic sites. The high R-value suggested that these differences are primarily due to compositional variation rather than dispersion alone.

### Structure and geographic patterns of nival bacterial communities across global sites

**Fig. 5:**
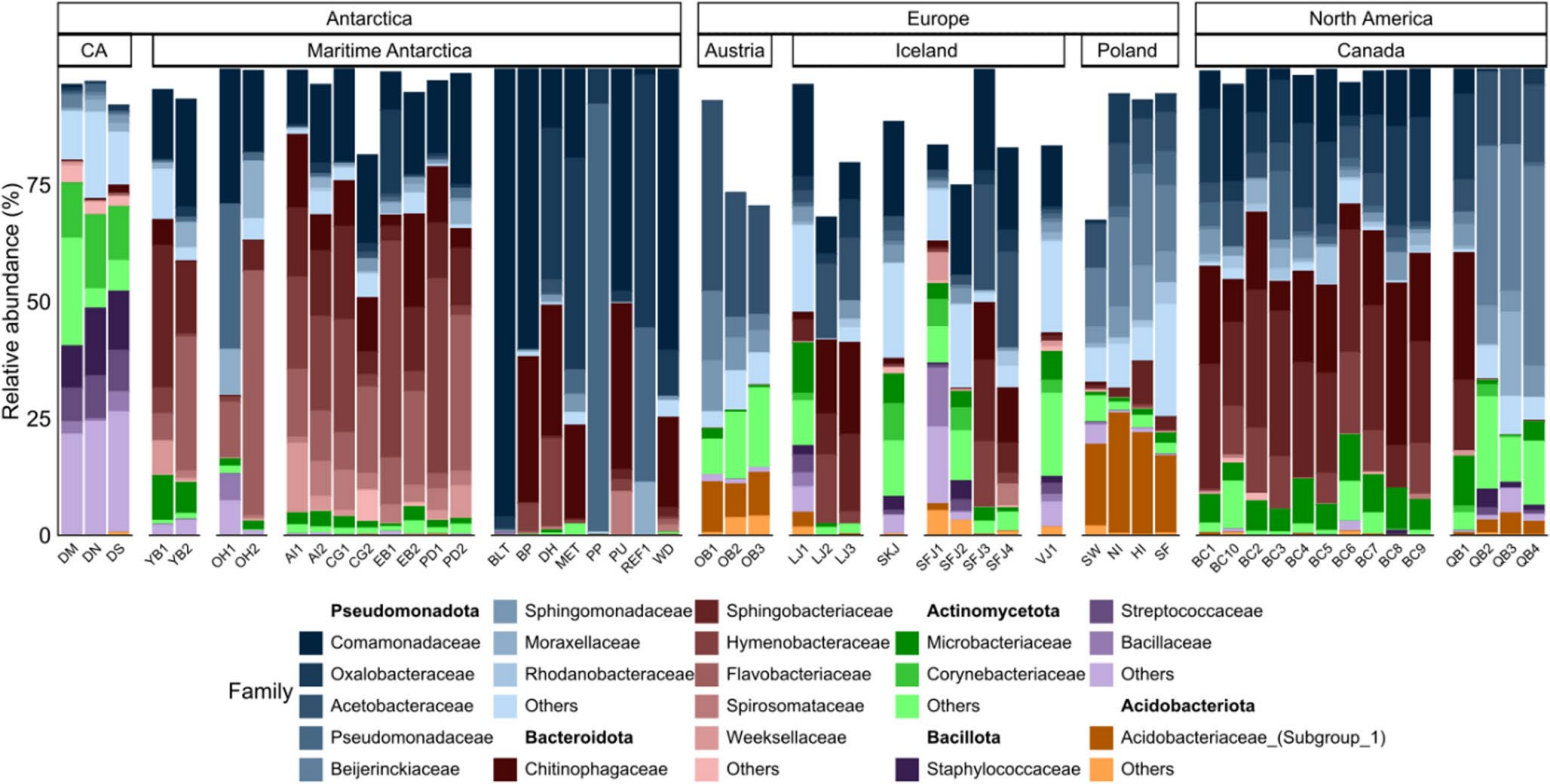
Taxonomic composition of the snow microbiome across global sites and regions, at the family level. CA: Continental Antarctica; AM: Maritime Antarctica. The specific names of the sites (x axis) are detailed in Table S1

Global snow-borne bacterial communities were diverse, encompassing 40 assigned phyla. Among these, Pseudomonadota was the most abundant (45%), followed by Bacteroidota (29%). Other less abundant phyla included Actinomycetota (8%), Acidobacteriota (6%) and Bacillota (4%). A total of 509 bacterial families were identified, and 56 ASVs remained unclassified at levels below Order and Phylum, collectively contributing to a minor fraction of the dataset (4% of ASVs). The most abundant families accounted for nearly 68% of all sequences in Antarctic snow samples, with Sphingobacteriaceae (11%), Comamonadaceae (10%), Chitinophagaceae (8%) and Oxalobacteraceae (8%) being the most prevalent. Acetobacteraceae (7%), Beijerinckiaceae (6%), and Hymenobacteraceae (5%) accounted for a small percentage of the dataset. The distribution of these families across the sampled sites is presented in Fig. 5.

The Antarctic snow microbiome exhibited heterogeneity across different geographical locations, without a clear microbial pattern. Sampling sites in Martel Inlet showed a high abundance of Comamonadaceae, Pseudomonadaceae, and Oxalobacteraceae, which together accounted for 75% of the total microbial community. Other Maritime Antarctic regions, such as Fildes Peninsula, O’Higgins, and Doumer Island, were dominated by Bacteroidota families, although exhibiting distinct patterns: Hymenobacteraceae was more prevalent in Fildes Peninsula (20%), Flavobacteriaceae in O’Higgins (26%), and Sphingobacteriaceae in Doumer Island (31%). While some differences in bacterial family abundances were subtle across sites, a distinct representation of families was observed within the Maritime Antarctica. Antarctic Plateau sites exhibited a completely different bacterial composition from Maritime Antarctic sites, with higher abundances of Corynebacteriaceae (13%), Staphylococcaceae (13%), and Streptococcaceae (9%). Additionally, samples from the Antarctic continent displayed higher overall diversity, as indicated by a greater proportion of sequences classified as “Other,” suggesting the presence of numerous bacterial groups with abundances below 1%. Rare bacterial families (< 1% relative abundance) were identified across Antarctic snow samples and visualized in a heatmap (Figure S1). These taxa showed a heterogeneous distribution among samples, with a higher occurrence of rare families in Continental Antarctica compared to Maritime Antarctic sites.

European sites exhibited more diverse snow bacterial communities compared to Antarctic and North America sites, with a broader representation of families from the phyla Pseudomonadota, Bacteroidota, and Actinomycetota. Within Europe, Iceland stood out for its higher abundance of Bacillota (Staphylococcaceae) and Actinomycetota (Corynebacteriaceae), while Poland and Austria showed increased representation of Acidobacteriota and Acetobacteriaceae, respectively. In contrast, the Canadian sites displayed community compositions more similar to those of Maritime Antarctica, both being dominated by families within Bacteroidota and Pseudomonadota.

The presence of a potential snow core microbiome was evaluated across all sampling locations based on taxon prevalence. Herein, a strict core microbiome was defined as the assemblage of taxa detected in 100% of the samples, using a minimum detection threshold of 0 or 0.1% (0.001) relative abundance. This analysis was conducted at both genus and family levels. Under these criteria, no bacterial taxa were detected in all samples at either taxonomic level, indicating the absence of a strict core microbiome shared across the studied locations.

### Taxa with differential abundance across snow-covered regions

**Fig. 6:**
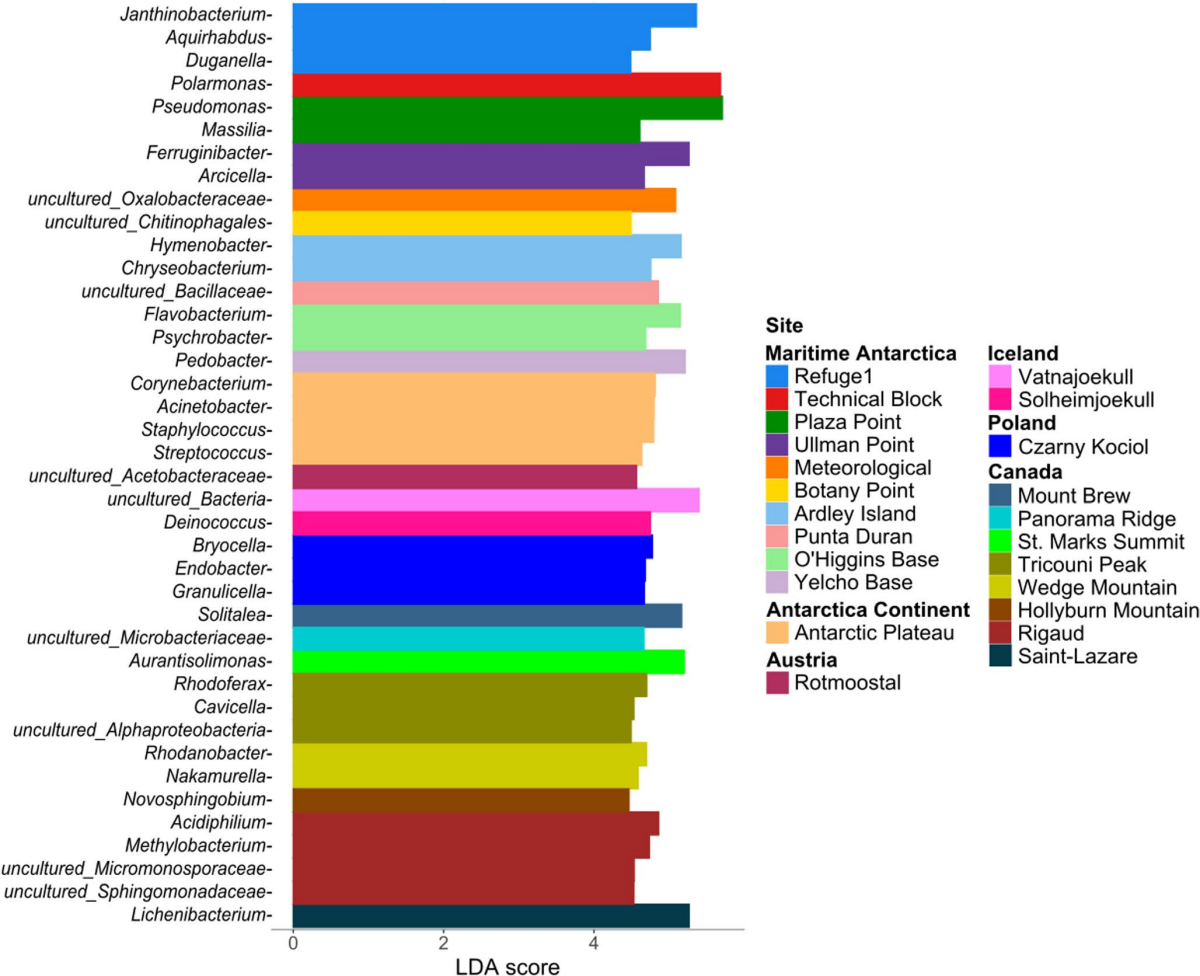
LEfSe analysis showing the bacterial genera with significant differential abundance among sites. Genera with longer bars have a greater contribution in distinguishing geographic regions. Colors represent sites grouped by country or region

An effect size analysis using Linear Discriminant Analysis (LEfSe) was conducted to identify the taxa that contributed most to the dissimilarities among snow microbiomes. A threshold of 3 was applied to determine the magnitude of differences in taxon abundance, allowing for the identification of bioindicator genera (Fig. 6). The Antarctic Plateau and Rigaud sites exhibited the highest number of differentially abundant groups, with some taxa at the Rigaud site remaining unclassified at the genus level. Genera such as *Pseudomonas* and *Polaromonas* had the highest LDA scores (> 5.8) and were differentially abundant at Plaza Point and Technical Block, respectively. These taxa dominate the microbiomes of their respective sites and help explain the observed bacterial community differences.

### Co-occurrence patterns of snow microbiomes

Co-occurrence networks were constructed for most of the sites analyzed in this study to infer the interactions among species within microbial communities. Due to sampling limitations, such as community size and lack of replicates, co-occurrence networks could not be constructed for the bacterial communities for most Martel Inlet sites (Refuge 1, Technical Block, Plaza Point, Wanda, Meteorological Point, Botany Point), Fildes Peninsula (EB2), British Columbia (BC2, BC6), all Quebec samples, and most Icelandic samples, except Langjoekull (LJ2) (Table S1). The main topological properties of the networks for each site are summarized in Table S3.

Across all regions, network architecture varied considerably in size, connectivity, and modular organization. Most networks exhibited moderate to high modularity (> 0.4), indicative of compartmentalized structures commonly associated with increased stability and ecological specialization. Several networks displayed high local clustering coefficients (> 0.5) and low average path length (< 3), consistent with “*small-world*” properties. Positive correlations predominated across nearly all networks, frequently exceeding 90% of total interactions, suggesting that snow microbiomes are largely structured by shared environmental responses or cooperative associations.

In the Maritime Antarctica, networks generally showed variability in network size. Martel Inlet (DH and PU), O’Higgins (OH1 and OH2), and Yelcho Base (YB1 and YB2) sites displayed low average path length (< 3) and a predominance of positive correlations. Most networks also exhibited moderate modularity (generally > 0.45), indicating recurrent compartmental organization across sites. In contrast, structural heterogeneity was evident within the Fildes Peninsula. While some networks (AI1, AI2, CG1) were characterized by smaller size (fewer nodes and edges), others (CG2, EB1, and PD1) displayed larger and more connected architectures, reflected by higher numbers of nodes and greater average degree. PD2 exhibited a distinct structural profile, with high modularity (0.63), 12 detected modules, elevated average path length (> 4), and the highest proportion of negative interactions (22%). Continental Antarctic networks (DM, DN, and DS) were larger and more interconnected than those from the Maritime Antarctica, exhibiting very high average degree and clustering coefficients. DM and DN showed low average path length (< 3), indicating compact network structure, whereas DS presented a higher average path length (> 6), high clustering, and modularity, indicating a structurally compartmentalized and potentially robust network.

European and North American networks showed a structural profile characterized by moderate to high modularity, high connectivity in several sites, and a predominance of positive correlations. Austrian networks represented some of the largest and most densely connected European systems. In contrast, British Columbia encompassed both highly modular (e.g., BC4, BC9) and comparatively sparse networks (e.g., BC1, BC3). Within Europe, Polish sites displayed marked heterogeneity, while the Icelandic network (LJ2) was distinguished by comparatively elevated centralized betweenness.

Topological parameters of individual ASVs were used to infer ecological roles based on within-module connectivity (Zi-score) and among-module connectivity (Pi-score). Peripheral nodes (low Zi and Pi values) represent taxa with few connections to other taxa within their modules and are often considered specialists with limited interactions in the network (Guimerà and Nunes Amaral 2005). Connector nodes (low Zi and high Pi) link different modules and may facilitate inter-module interactions. Module hubs (high Zi and low Pi) correspond to ASVs with a high number of connections within their own modules. The distribution of these ecological roles and the taxonomic affiliation of the nodes across the different sites are shown in the Zi–Pi plot (Figure S2, Supplementary Material).

Table 1 summarizes the ecological classification of nodes across the different snow ecosystem networks analyzed. Most nodes in the networks were classified as peripheral, with several networks composed exclusively of this ecological category. Connector nodes were detected mainly in networks from Fildes Peninsula, British Columbia, and Poland, indicating potential links between modules in these locations. Module hubs were also identified in some of these networks, suggesting the presence of highly connected taxa within specific modules. No network hubs (high Zi and high Pi) were detected in any of the analyzed networks.

**Table 1:** Ecological classification of nodes based on within-module (Zi) and among-module (Pi) connectivity across the co-occurrence networks of the snow ecosystems. Nodes were categorized as peripheral nodes, connectors, module hubs, or network hubs according to their topological roles

Local	Site	Peripheral nodes	Connectors	Module hubs	Network hubs
Martel Inlet	DH	25	-	–	–
Martel Inlet	PU	7			–
Antarctic Plateau	DM	788			–
Antarctic Plateau	DN	755			–
Antarctic Plateau	DS	764			–
Fildes Peninsula	AI1	57	1	1	–
Fildes Peninsula	AI2	9	4		–
Fildes Peninsula	CG1	29			–
Fildes Peninsula	CG2	135		3	–
Fildes Peninsula	EB1	173		2	–
Fildes Peninsula	PD1	88		1	–
Fildes Peninsula	PD2	86	4		–
Yelcho Base	YB1	63		1	–
Yelcho Base	YB2	30			–
O’Higgins	OH1	13			–
O’Higgins	OH2	40			–
British Columbia	BC1	26			–
British Columbia	BC3	29			–
British Columbia	BC4	93			–
British Columbia	BC5	29	4		–
British Columbia	BC7	19	9		–
British Columbia	BC8	40		1	–
British Columbia	BC9	117			–
British Columbia	BC10	110		2	–
Austria	OB1	582		8	–
Austria	OB2	798		9	–
Austria	OB3	1075		11	–
Poland	SW	243	4	1	–
Poland	SF	45	1		–
Poland	HI	316	1	1	–
Iceland	LJ2	139			–

### Influence of environmental variables

Based on monthly average climate data from the ERA5-Land model for the geographic coordinates of the sites analyzed, distinct climatic profiles were identified across the regions **(Table ****S1****)**. The Antarctic Plateau, exhibited the highest wind velocities and extremely low average temperatures (ranging from − 17 to −11 °C). Martel Inlet sites (e.g., Refuge1, Technical Block, Plaza Point) and stations like O’Higgins and Yelcho recorded high monthly snow precipitation averages (> 1.5 mm), being the highest ones (> 10–20 mm) observed in British Columbia (Sky Pilot Mountain, Tricouni Peak, Hollyburn Mountain, and Mount Seymour) and in the sites Ullman Point, Wanda and Botany Point, in the Martel Inlet.

Sites in Austria, Canada (Quebec), and Iceland (Langjoekull, Skaftafelljoekull, and Solheimjoekull), sampled in winter, showed low average air temperatures (− 14 to − 5 °C), high snow precipitation (> 1.5 mm), and minimal snowmelt. Some British Columbia sites (Hollyburn Mountain, Mount Seymour, Sky Pilot Mountain) and all Polish sites were sampled in spring, while two Quebec sites were sampled in autumn. Despite these seasonal differences, they exhibited similar climatic conditions, with temperatures ranging from − 3 to 0 °C, low snow precipitation, and limited snowmelt. Most sites in British Columbia and Iceland were sampled in summer and generally exhibited low monthly averages of snow precipitation and snowmelt. Notably, British Columbia sites recorded the highest monthly solar radiation values (> 9.9 MJ/m²).

**Fig. 7:**
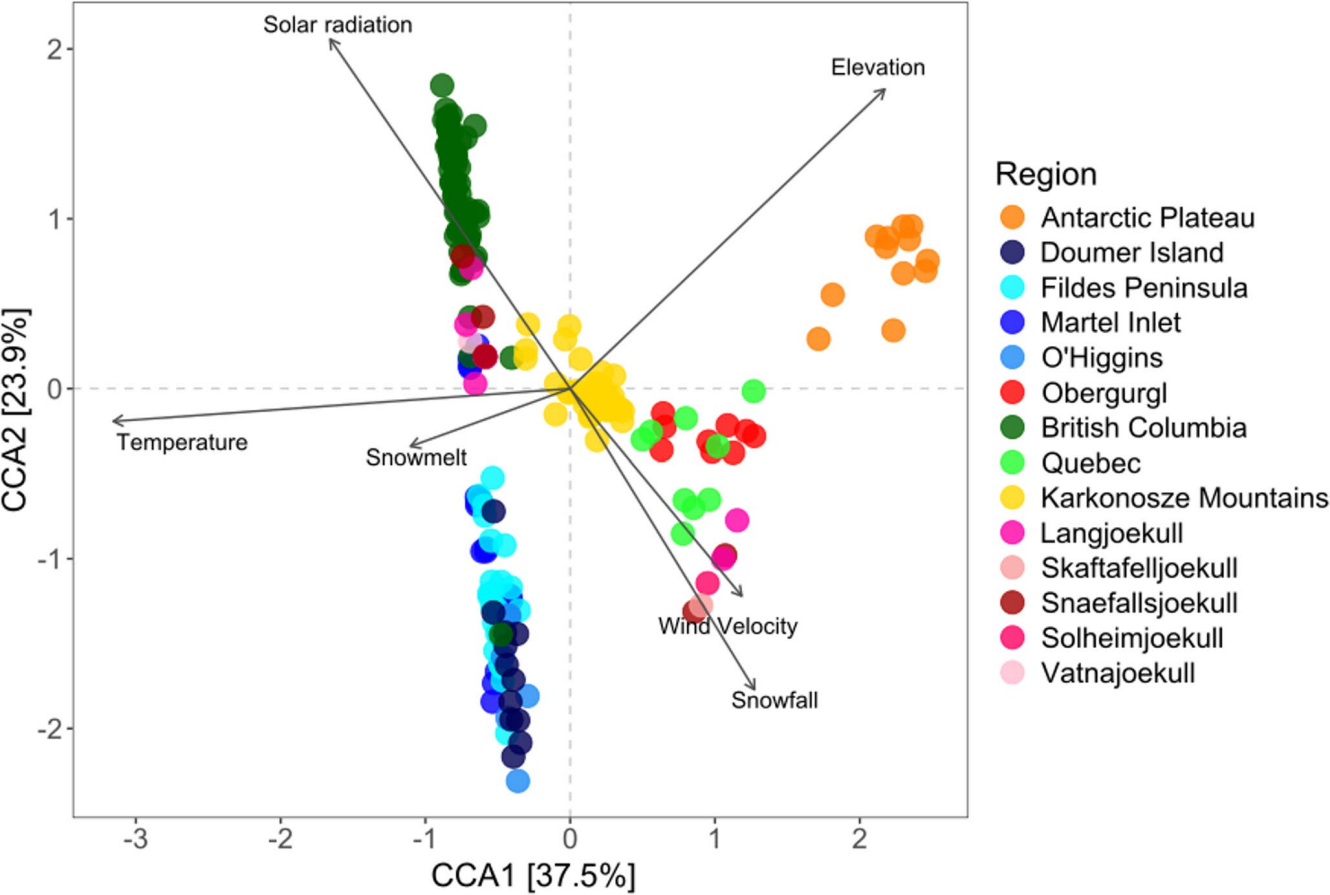
Canonical Analysis (CCA) plot showing the relationships between bacterial community composition (colored circles) and environmental variables (black arrows). The direction and length of the arrows indicate the strength and direction of environmental gradients. Sampling regions are color-coded and grouped by geographic origin: Antarctic Plateau (Continental Antarctica); Doumer Island, Fildes Peninsula, Martel Inlet, and O’Higgins (Maritime Antarctica); Obergurgl (Austria); British Columbia and Quebec (Canada); Karkonosze Mountains (Poland); Langjökull, Skaftafellsjökull, Snæfellsjökull, Sólheimajökull, and Vatnajökull (Iceland)

The first two canonical axes explained 61.4% of the constrained variation in bacterial community composition (CCA1 = 37.5%; CCA2 = 23.9%). CCA1 was primarily structured by temperature and solar radiation, which showed strong correlations with the main ordination gradient, whereas CCA2 reflected additional variation associated with wind velocity and snowfall. Marginal permutation tests indicated that wind velocity (*p* = 0.001) presented the highest independent contribution to community variation, followed by snowfall (*p* = 0.001), solar radiation (*p* = 0.001), temperature (*p* = 0.002), and elevation (*p* = 0.005), whereas snowmelt did not show a significant effect (*p* = 0.587). The high altitude of the Antarctic Plateau was strongly associated with the distinct bacterial community composition observed at these continental sites. Maritime Antarctica sites (e.g., Fildes Peninsula, Martel Inlet, Doumer Island) clustered closely and were associated with higher temperatures and snowmelt, reflecting milder climatic conditions and seasonal melt influence during summer. Solar radiation was highest at the British Columbia sites and was the most strongly correlated environmental factor influencing bacterial community composition. Wind velocity and snowfall were associated with samples from Iceland, Austria, and Quebec (predominantly collected in winter), suggesting that these variables play an ecologically significant role in shaping bacterial communities in these regions during the cold season Fig. 7.

**Fig. 8:**
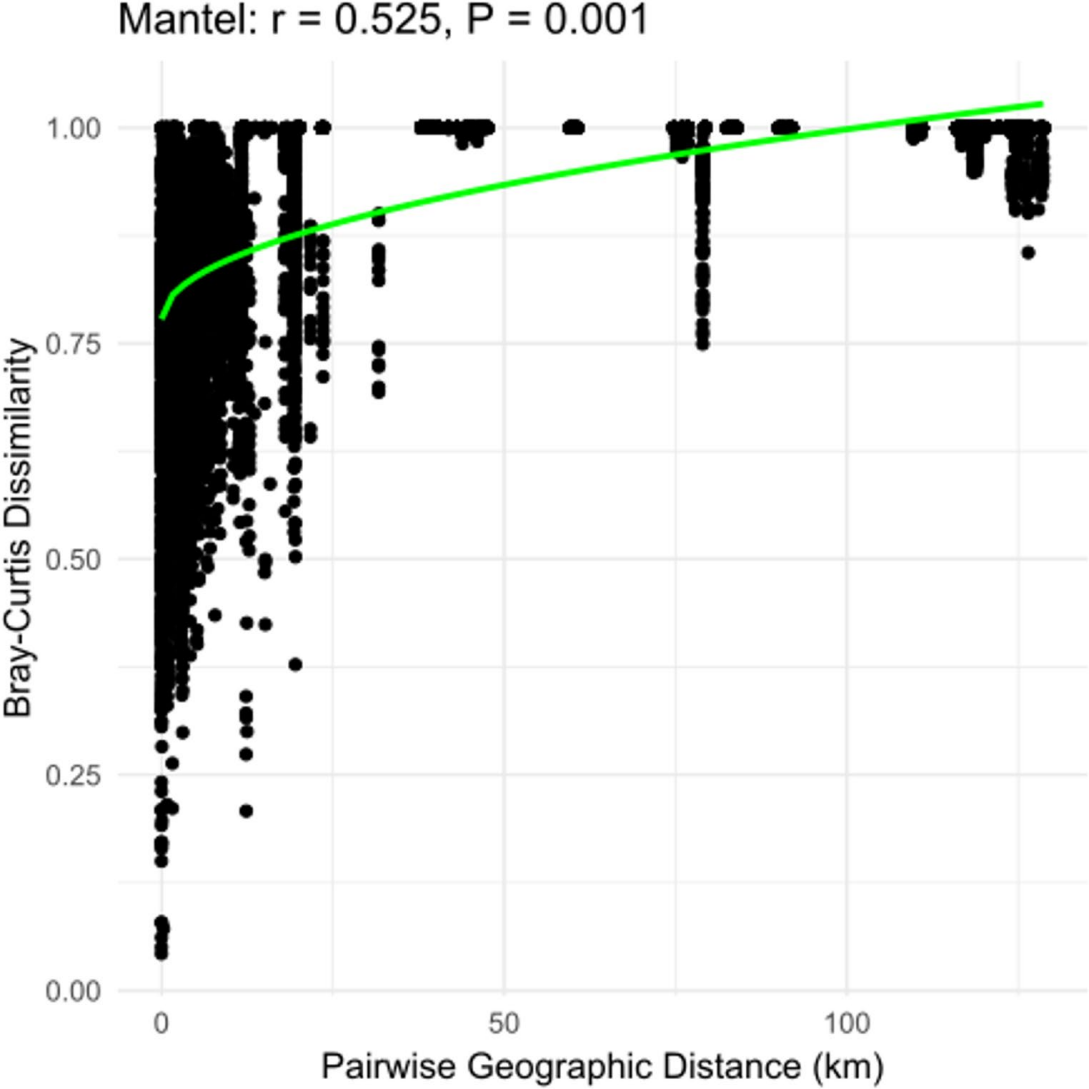
Regression analysis and Mantel test of pairwise geographic distance (km) against iterative Bray-Curtis dissimilarity values for bacterial communities across snow sites. Both the regression analysis and Mantel test indicate that the further apart two samples are from each other, the more dissimilar the bacterial communities are

The Mantel test revealed significant correlations between environmental variables and bacterial community composition across all sites. Solar radiation (*r* = 0.387, *p* = 0.001) and snowfall (*r* = 0.364, *p* = 0.001) showed the strongest associations, suggesting that light availability and snow accumulation play key roles in shaping bacterial diversity. Elevation (*r* = 0.337, *p* = 0.001) and temperature (*r* = 0.281, *p* = 0.001) were also influential, indicating that altitude-driven climatic changes impact bacterial distribution. Although snowmelt (*r* = 0.060, *p* = 0.004) was significantly correlated, their weaker coefficient suggests a less pronounced but still relevant effect. Additionally, the distance-decay relationship was significant, with bacterial community dissimilarity increasing as geographic distance increased (Mantel *r* = 0.525, *p* = 0.001), supporting the isolation-by-distance (IBD) hypothesis **(**Fig. 8**)**. Linear regression analysis further supported this pattern (R² = 0.275, *p* < 0.001), indicating that dispersal limitation contributes to bacterial differentiation. These results highlight the importance of climate-related variables and spatial distance in structuring snow bacterial communities across different latitudes, reinforcing the idea that environmental selection and dispersal limitation are key drivers of microbial diversity in polar and alpine ecosystems.

## Discussion

The bacterial communities inhabiting snow are taxonomically diverse and exhibit complex community structures shaped by environmental and geographic factors. Our findings revealed significant heterogeneity in the composition and diversity of globally distributed snow microbiomes, with distinct patterns emerging across geographic locations.

In this study, Antarctic samples were obtained from both Continental Antarctica (Antarctic Plateau) and Maritime Antarctica (Martel Inlet, Fildes Peninsula, and the O’Higgins and Yelcho stations). A clear distinction in bacterial composition and phylogenetic distance was observed between the Antarctic Plateau and Maritime Antarctica sites. Bacterial communities from the Antarctic Plateau exhibited higher overall bacterial diversity, including a greater contribution of taxa belonging to the phyla Bacillota and Actinomycetota. Previous studies have reported that microbial communities in Antarctic snow and ice frequently include stress-tolerant bacterial groups such as Bacillota, Actinomycetota, and Pseudomonadota, which are well adapted to cold, oligotrophic, and highly irradiated environments (Doytchinov and Dimov 2022; Michaud et al. 2014). According to Parro et al. (2025), snow and ice layers in the Antarctic Plateau can act as reservoirs of airborne microorganisms, allowing the accumulation of taxa adapted to extreme cold and hyperarid conditions. In contrast, Maritime Antarctic sites showed greater variability in community composition among locations. Our findings suggest a stronger influence of local environmental drivers, such as higher snowfall, milder temperatures, and seasonal snowmelt processes, which can create dynamic environmental conditions that shape microbial communities (Antony et al. 2016; Garnica et al. 2025). Previous studies in Maritime Antarctic snowpacks have also shown that bacterial community composition can be influenced by environmental drivers including snowmelt dynamics, temperature, wind patterns, and atmospheric deposition (Hodson et al. 2021; Malard et al. 2019). Although Maritime Antarctic environments generally harbor diverse microbial communities, among the Antarctic sites analyzed here, Martel Inlet showed lower richness and diversity compared to the other Antarctic sites, along with the dominance of specific bacterial phyla. Previous studies suggest that the geographic setting of Martel Inlet, a semi-enclosed fjord within Admiralty Bay (King George Island), subjects the area to pronounced temperature and salinity gradients, which can affect bacterial diversity (Fortunato and Crump 2015; Gutiérrez et al. 2015). Nonetheless, the bacterial community at Martel Inlet was dominated by members of the phyla Pseudomonadota and Bacteroidota (notably Chitinophagaceae and Flavobacteriaceae), which are commonly found in fjord environments at high latitudes (Gutiérrez et al. 2015; Zeng et al. 2013).

The Antarctic Plateau sites exhibited the highest bacterial diversity, surpassing not only the Maritime Antarctic sites but also most alpine nival environments. Snow samples from the Antarctic Plateau showed relatively similar bacterial community profiles to those from Austria, Iceland, and Quebec (QB1–QB4), particularly regarding the high abundance of Bacillota families such as Staphylococcaceae, Streptococcaceae, and Bacillaceae, as well as several representatives of Actinomycetota. These groups were consistently more abundant in these sites compared to others. This convergence may reflect shared environmental pressures, such as extreme cold temperatures, oligotrophic snow, and high UV exposure, which could select for stress-tolerant, spore-forming, and radiation-resistant bacteria (Cui et al. 2023). These findings suggest that climatic and environmental factors are key drivers of nival microbiome composition, while spatial distance also contributes to community differentiation across regions (Nigro et al. 2024; Qi et al. 2023; Vuille 2011; Wake 2014). Moreover, samples from Maritime Antarctica clustered with those from British Columbia (Canada), indicating a similar bacterial composition at both sites. In both regions, the bacterial communities were associated with snow algae blooms. Although several studies have explored the relationship between bacteria and algal species, no definitive ecological association has been established (Soto et al. 2022; Yakimovich et al. 2020). The similarity in bacterial composition between Maritime Antarctica and British Columbia is likely due to the higher temperature (summer season) and extensive snowmelt, which releases nutrients and carbon sources stored in snow and ice (Capotondi et al. 2024; Zardi 2024).

Although similarities in bacterial composition were observed among some snow environments, our analyses did not identify a universal core microbiome across the studied sites. This result suggests that nival microbial communities are highly context-dependent and shaped by a combination of environmental and geographic factors. Mantel analyses and regression models further indicated that spatial distance, together with environmental variables, contributes to structuring bacterial communities in snow ecosystems. These patterns are consistent with previous studies showing strong spatial variability in Antarctic snow microbial communities, where community composition changes with geographic location and environmental inputs (Brown and Jumpponen 2019; Courville et al. 2020; Malard et al. 2019). While climatic and environmental conditions exert selective pressure on microbial community composition, seasonal variation did not appear to be a dominant driver in this study. These patterns are also supported by the LEfSe analysis, which revealed distinct sets of differentially abundant bacterial taxa associated with specific regions and sampling sites, reinforcing the idea that local environmental conditions and geographic separation contribute to the differentiation of nival microbiomes.

Peripheral nodes represented the vast majority of taxa across the snow microbial networks analyzed in this study, whereas connectors and module hubs occurred much less frequently and no network hubs were detected. Peripheral taxa typically represent weakly connected species that interact with only a limited number of partners within the network (Olesen et al. 2007). The predominance of peripheral nodes suggests that snow microbial communities are largely structured by strong environmental filtering rather than by highly integrated ecological interactions. In contrast, connectors and module hubs, which contribute to network cohesion and stability (Banerjee et al. 2018), were relatively rare, particularly in Antarctic sites, indicating a lower degree of network integration in these extreme environments. Similar results were observed in studies with glacier surface snow in Tibetan Plateau (Chen et al. 2021) and permafrost (Zhang et al. 2023).

The bacterial networks of Maritime Antarctica (Martel Inlet, Fildes Peninsula, O’Higgins and Yelcho Base) exhibited characteristics associated with more dynamic systems and potentially greater environmental stress. These networks showed a higher proportion of negative interactions, likely associated with competition for resources or unstable environmental conditions (Coyte et al. 2015; Faust and Raes 2012), as well as elevated betweenness centrality, suggesting a stronger dependence on keystone taxa and therefore greater vulnerability to the loss of structural connectors (Banerjee et al. 2018; Berry and Widder 2014). Similar network structures have been reported in other cold environments, including surface snow from Tibetan glaciers (Chen et al. 2021), glacial lakes (Modenutti et al. 2023), and cold steppe ecosystems (Yang et al. 2024), where microbial networks tend to show lower density, moderate modularity, and relatively simplified interaction patterns.

The alpine networks in British Columbia showed a similar profile in terms of the topological metrics of the networks. Although British Columbia is not a marine area, the sampled sites correspond to alpine environments located in a coastal region (Yakimovich et al. 2020). In polar and alpine ecosystems, coastal areas generally show greater environmental variability due to seasonal thawing, higher incidence of ultraviolet radiation, as well as fluctuations in humidity and temperature (Jansson and Tas 2014; Nemergut et al. 2007). The fluctuation of these environmental stressors tend to destabilize microbial networks, promoting an increase in the frequency of competitive interactions and greater dependence on key taxa (Banerjee et al. 2018; Faust and Raes 2012). In addition, the samples analyzed were collected during spring and summer - periods marked by intense environmental transitions - which contributes to the formation of dynamic and structurally less cohesive microbial communities (Rime et al. 2016). Environments with high variability create distinct and unstable ecological niches, favoring the existence of connector taxa that facilitate stability and communication between different microbial modules to maintain the functionality of the community. These characteristics give these microorganisms a central role in maintaining the stability and resilience of microbial networks, acting as bridges that connect different ecological modules (Faust and Raes 2012).

The bacterial networks of continental Antarctica and of the continental alpine regions (Austria, Poland and Iceland) revealed a structure distinct from that observed in coastal environments. These systems presented broad, densely connected networks with a high degree of compartmentalization, reflecting a functional organization strongly shaped by environmental stability and the spatial heterogeneity typical of cold, continental ecosystems (Zhang et al. 2023). The greater connectivity within modules, in contrast to the lower connectivity between them, suggests functionally organized communities in relatively cohesive and possibly specialized cores, which is consistent with patterns observed in cryoconites, permafrost soils, and deep glacial ecosystems, where microbial interactions tend to be intense but more restricted to adapted functional groups (Zhang et al. 2023).

Modular structure and niche specialization appear to be recurrent organizational strategies in nival bacterial communities from the Antarctic Plateau and alpine regions such as Austria, Iceland, and Poland, a pattern also reported in cold environments including alpine pastures (Xiao et al. 2025), glacial streams (Ren and Gao 2019) and Antarctic soils (Savaglia et al. 2024). The majority of the module hubs identified in this study were concentrated in samples from Poland and Austria. This distribution is consistent with the modular structure observed in the networks from these regions, characterized by moderate compartmentalization and high intra-module connectivity. Studies conducted in Arctic tundra (Wong et al. 2023) and glacier forefield streams and soils (Ren and Gao 2019) indicate that the higher frequency of module hubs in networks with intermediate modularity and local environmental diversity reinforces the role of these taxa in the structural organization and resilience of microbial communities in cold, heterogeneous, and ecologically compartmentalized zones (Banerjee et al. 2018; Olesen et al. 2007).

Together, these results indicate that the structure of microbial interaction networks in snow ecosystems is strongly influenced by environmental stability and variability, with dynamic coastal environments favoring simpler and more competitive networks, whereas more stable continental environments promote modular and specialized microbial assemblages.

## Conclusions

This study addressed two key hypotheses: (1) microbial diversity in Antarctica is lower than in temperate snow-covered regions due to extreme isolation and environmental factors such as low temperature, high elevation, and differences in snow accumulation and melt dynamics; and (2) microbial communities in snow vary significantly along latitudinal gradients, both regionally within Antarctica and globally, when comparing Antarctic and non-Antarctic sites.

Our findings support only the second hypothesis. An overall high bacterial richness and diversity was observed in both Antarctic regions, Continental and Maritime, except for the Martel Inlet, a geographically isolated site, which showed reduced bacterial richness and diversity. When comparing regions across latitudes, snow bacterial communities in the Alps, Quebec, and Iceland shared notable similarities, likely driven by overlapping environmental factors such as snow conditions, temperature range, and seasonal dynamics. These findings suggest that seasonal and atmospheric parameters play a significant role in shaping snow bacterial communities, irrespective of geographic distance. Understanding these patterns is particularly important given the ecological role of snow-associated microbial communities within the cryosphere.

Microbial communities inhabiting snow environments represent an important component of the cryosphere and may influence nutrient cycling and ecosystem functioning in polar regions. As climate change accelerates warming in high-latitude environments, alterations in snow cover dynamics and melt patterns may affect microbial community composition and ecological interactions. In this scenario, keystone taxa identified in microbial networks may play a pivotal role in maintaining community structure and resilience under changing environmental conditions. Together, these findings highlight how geographic location, atmospheric processes, and environmental conditions shape snow microbial communities across hemispheres and contribute to the diversity patterns observed in cold ecosystems.

## Supplementary Information

Below is the link to the electronic supplementary material.

Supplementary Material 1 (DOCX 63.7 KB)

Supplementary Material 2 (DOCX 246 KB)

## Data Availability

Additionally, the sequencing data generated in this study have been deposited in the NCBI Sequence Read Archive under BioProject accession number PRJNA1438192.
