# Supplementary material for: Disentangling bacterial diversity and biogeography in snow-covered regions
Source: World J Microbiol Biotechnol. 2026 Apr 28;42(5):242. doi: 10.1007/s11274-026-04918-w (PMC13121194; doi:10.1007/s11274-026-04918-w)
Supplement: Supplementary file 1 — Supplementary Material 1 (DOCX 63.7 KB) [file 11274_2026_4918_MOESM1_ESM.docx]

**Supplementary Material**

# **Disentangling Bacterial Diversity and Biogeography in Snow-Covered Regions**

Jessica Bianca da Silva^a, b^, Paulo Eduardo Aguiar Saraiva Câmara ^c^, Luiz Henrique Rosa ^d^, Valéria Maia de Oliveira ^a, b^

**a** *Microbial Resources Division, Research Center for Chemistry, Biology and Agriculture (CPQBA), State University of Campinas, Paulínia, SP, CEP: 13081-970, Brazil*

**b** *Institute of Biology, State University of Campinas, Campinas, SP, CEP: 13083-862, Brazil*

**c** *Department of Botany, University of Brasilia – UNB, Brasilia,*

**d** *Institute of Biological Sciences, Federal University of Minas Gerais - UFMG, Belo Horizonte, MG, CEP 31270-901, Brazil*

* Corresponding author: Division of Microbial Resources, Research Center for Chemistry, Biology and Agriculture (CPQBA), UNICAMP, Av. Alexandre Cazelatto, 999, Betel, Zip code 13148-218, Paulínia, São Paulo, Brazil. Tel: +55 (19) 2139-2874 E-mail address: jbd.silva@outlook.com


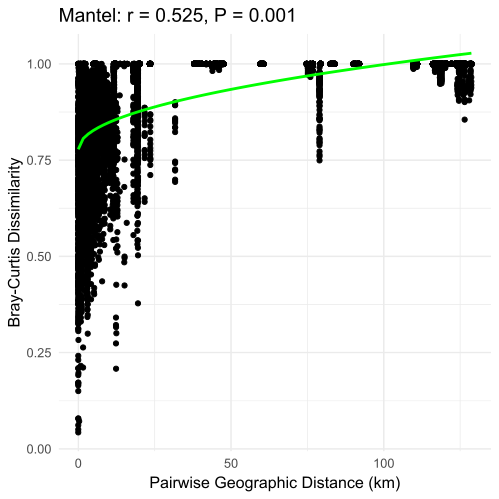


**Figure S1.** Regression analysis and Mantel test of pairwise geographic distance (km) against iterative Bray-Curtis dissimilarity values for bacteria across snow sites. Both the regression analysis and Mantel test indicate that the further apart two samples are from each other, the more dissimilar the bacteria communities are.
