# Supplementary material for: Disentangling bacterial diversity and biogeography in snow-covered regions
Source: World J Microbiol Biotechnol. 2026 Apr 28;42(5):242. doi: 10.1007/s11274-026-04918-w (PMC13121194; doi:10.1007/s11274-026-04918-w)
Supplement: Supplementary file 2 — Supplementary Material 2 (DOCX 246 KB) [file 11274_2026_4918_MOESM2_ESM.docx]

**Supplementary Material**

# **Disentangling Bacterial Diversity and Biogeography in Snow-Covered Regions**

Jessica Bianca da Silva^a, b^, Paulo Eduardo Aguiar Saraiva Câmara ^c^, Luiz Henrique Rosa ^d^, Valéria Maia de Oliveira ^a, b^

**a** *Microbial Resources Division, Research Center for Chemistry, Biology and Agriculture (CPQBA), State University of Campinas, Paulínia, SP, CEP: 13081-970, Brazil*

**b** *Institute of Biology, State University of Campinas, Campinas, SP, CEP: 13083-862, Brazil*

**c** *Department of Botany, University of Brasilia – UNB, Brasilia,*

**d** *Institute of Biological Sciences, Federal University of Minas Gerais - UFMG, Belo Horizonte, MG, CEP 31270-901, Brazil*

* Corresponding authors: Division of Microbial Resources, Research Center for Chemistry, Biology and Agriculture (CPQBA), UNICAMP, Av. Alexandre Cazelatto, 999, Betel, Zip code 13148-218, Paulínia, São Paulo, Brazil. Tel: +55 (19) 2139-2874 E-mail address: [jbd.silva@outlook.com](mailto:jbd.silva@outlook.com); [vmaia@unicamp.br](mailto:vmaia@unicamp.br)


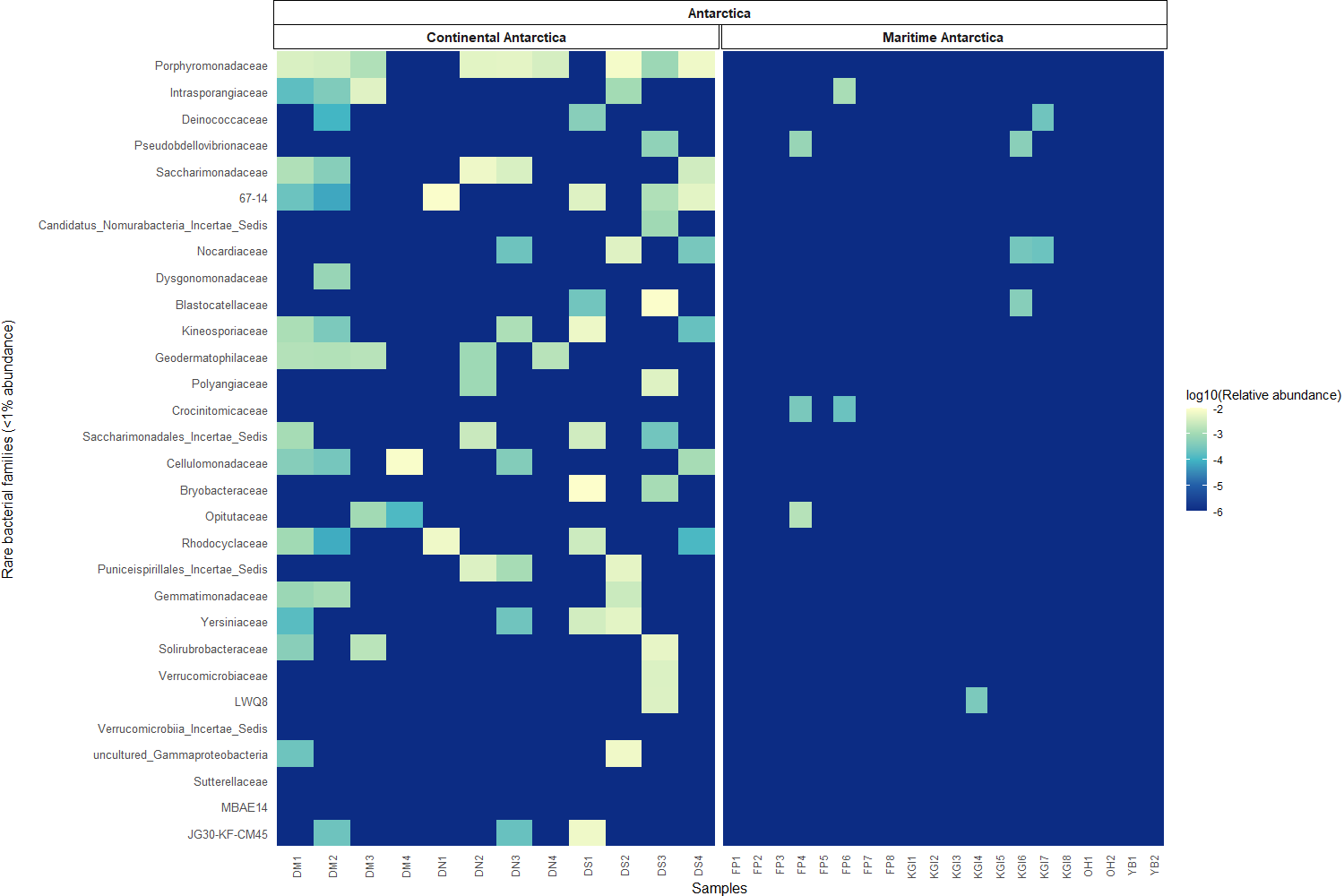


**Figure S1**. Heatmap showing the distribution of rare bacterial families (relative abundance <1%) across Antarctic snow samples. Color intensity represents log10-transformed relative abundance, with darker blue indicating lower abundance and yellow indicating higher abundance within the rare microbiome. Samples are grouped into Continental Antarctica and Maritime Antarctica.


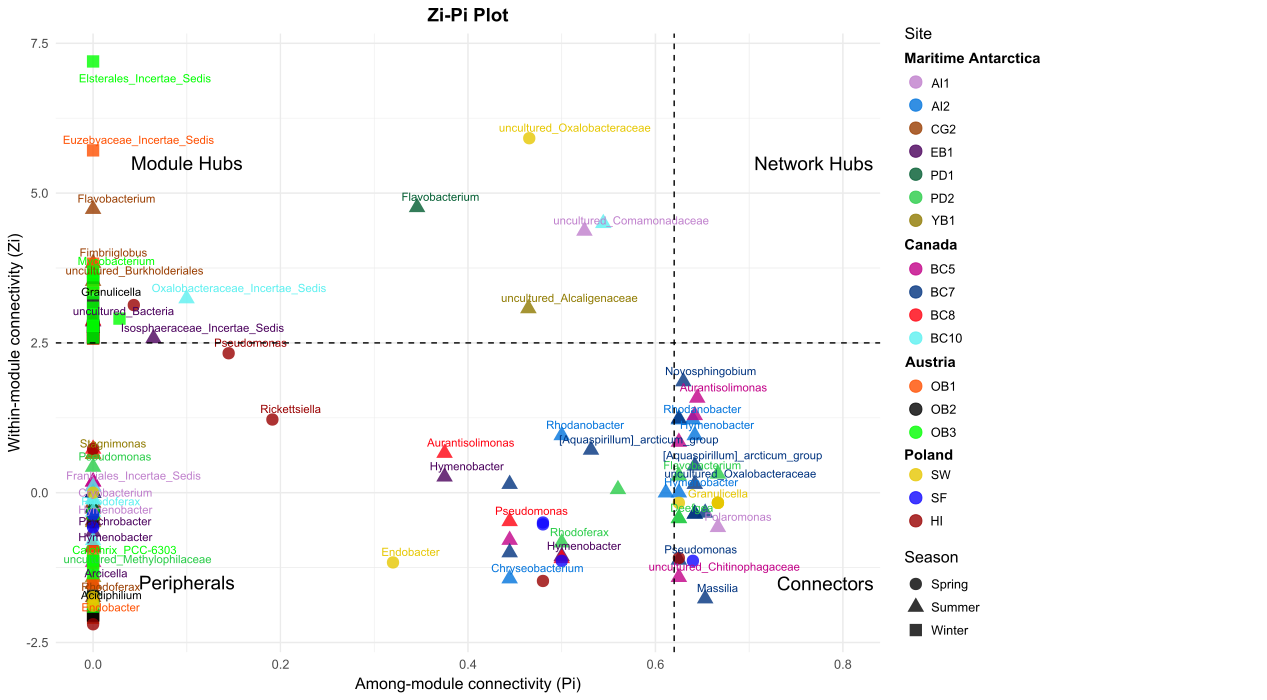


**Figure S2.** Identification of keystone taxa based on Zi-Pi plot showing the distributions of families based on their topological roles in the different areas and time samplings. Module hubs are identified as Zi-score ≥ 2.5 and Pi-score < 0.62; connectors are identified as Zi-score < 2.5 and Pi-score ≥ 0.62. Sampling regions are color-coded and grouped by geographic origin: Ardley Island (AI1 and AI2), Collins Glacier (CG2), Elefantera Beach (EB1), Punta Duran (PD1 and PD2) and Yelcho Base (YB1) located in Maritime Antarctica; British Columbia (BC5, BC7, BC8 and BC10) located in Canada; Rotmoostal (OB1, OB2 and OB3) located in Austria; Kociol Malego sites (SW, SF and HI) located in Poland.
